# Blood oxygen level-dependent MRI in patients with coronary artery disease and normal volunteers: a validation study against PET

**DOI:** 10.1186/1532-429X-11-S1-O37

**Published:** 2009-01-28

**Authors:** Theodoros D Karamitsos, Alejandro Recio-Mayoral, Jayanth R Arnold, Lucia Leccisotti, Paul Bhamra-Ariza, Ruairidh K Howells, Nick Searle, Matthew D Robson, Ornella E Rimoldi, Paolo G Camici, Stefan Neubauer, Joseph B Selvanayagam

**Affiliations:** 1 University of Oxford Centre for Clinical Magnetic Resonance Research (OCMR), Oxford, UK; 2 Medical Research Council (MRC) Clinical Sciences Centre, Imperial College of Science, Technology and Medicine, Hammersmith Hospital, London, UK

**Keywords:** Positron Emission Tomography, Myocardial Blood Flow, Normal Volunteer, Coronary Flow Reserve, Positron Emission Tomography Perfusion

## Background

Elevated deoxyhaemoglobin seen downstream in a territory subtended by a stenotic coronary artery can be assessed by blood oxygen level-dependent (BOLD) MRI. Deoxyhemoglobin is paramagnetic and acts as an intrinsic contrast agent leading to signal loss in T2- and T2*-weighted sequences. Previous animal and human BOLD studies at 1.5 Tesla using T2*-weighted sequences were fundamentally limited by the relatively small signal differences between normal and de-oxygenated myocardial regions. A new T2-prepared steady-state free precession (SSFP) BOLD sequence gave promising results in animal models at 1.5 Tesla.[1] In theory, implementation of this sequence at the higher field strength of 3 Tesla would further improve the detection of BOLD signal intensity (SI) changes. We sought to apply a T2-prepared SSFP BOLD sequence at 3 Tesla in patients with coronary artery disease (CAD) and normal volunteers, and validated it against perfusion measurements by Positron Emission Tomography (PET).

## Methods

Twenty-two patients (age 62 ± 8 yrs, 16 men) with CAD (at least 1 stenosis > 50% on quantitative coronary angiography-QCA) and 10 normal volunteers (age 52 ± 7 yrs, 7 men) underwent 3 T BOLD MRI and PET. For BOLD-CMR, a single mid-ventricular slice was acquired at mid-diastole using a T2-prepared SSFP pulse sequence with the following parameters: T2 preparation weighting 40 ms, matrix 168 × 192, slice thickness 8 mm, flip angle 44°. A set of 6 images was acquired at rest and at peak adenosine (140 μg/kg/min) stress. Using PET with oxygen-15 labeled water, myocardial blood flow (MBF) was measured at baseline and during adenosine hyperemia. The BOLD short-axis view was divided into 6 segments, according to the mid-ventricular segments of the 17-AHA segment model, and mean signal intensities (SI) were calculated using QMass (Medis) software. SI values were corrected for differences in T1-weighting owing to heart rate changes at stress and rest. PET images were analyzed with home-built software under MATLAB (MathWorks Inc.) and registered with the BOLD short-axis image using anatomical landmarks.

## Results

**Figure 1:**
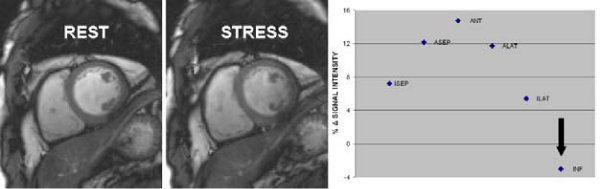
**An example of a patient with significant disease in the right coronary artery**. A SI drop was noted in the inferior wall (black arrow). All other myocardial segments showed a rise in SI during stress.

Based on the coronary anatomy, 59 myocardial segments were supplied by significantly stenosed vessels (stenosed segments) and 73 segments were supplied by vessels with minimal or no disease (remote to ischemia segments). A third group of myocardial segments (n = 60) from normal volunteers were labeled as 'normal' segments. Rest MBF, stress MBF, coronary flow reserve and BOLD-SI change of stenosed, remote to ischemia and normal segments are shown in Table 1. Taking QCA as the gold standard, cut-off values for stress MBF (≤ 2.45 ml/min/g – AUC 0.83) and BOLD SI change (≤ 3.74% – AUC 0.78) were determined to define ischemic segments. BOLD MRI and PET agreed on the presence or absence of ischemia in 18 of the 22 patients (82%), and in all normal subjects. With regards to per segment analysis: taking PET as the gold standard and by applying the cut-off values for stress MBF and BOLD SI, BOLD MRI had moderate sensitivity (63%) but very good specificity (88%) for the identification of ischemia. Minor off-resonance artifacts were found in 9 subjects (7 CAD patients and 2 normal volunteers). Figure 1 shows an example of a patient with significant disease in the right coronary artery.

**Table 1:** Rest MBF, stress MBF, coronary flow reserve (CFR) and BOLD-signal intensity (SI) change of stenosed, remote to ischemia and normal segments

	CAD patients		Normal volunteers	
	STENOSED N = 59	REMOTE N = 73	NORMAL N = 60	P-value
REST MBF (ml/min/g)	0.95 ± 0.03 (0.90 – 1.01)	0.94 ± 0.03 (0.88 – 0.99) ^†^	1.03 ± 0.03 (0.97 – 1.09)	0.04
REST MBF corrected [ml/min/g/(mmHg.bpm/10^4^)]	1.43 ± 0.05 (1.34 – 1.52)	1.42 ± 0.04 (1.34 – 1.51)	1.39 ± 0.04 (1.31 – 1.46)	0.76
HYPEREMIC MBF (ml/min/g)	2.11 (1.66 – 2.42) * ^‡^	2.73 (2.16 – 3.44)^†^	3.69 (3.16 – 4.61)	<0.001
CFR	2.31 (1.70 – 2.91) * ^‡^	3.00 (2.31 – 3.98)	3.62 (2.89 – 4.93)	<0.001
CFR corrected	1.41 (1.06 – 2.00) * ^‡^	2.01 (1.55 – 2.50)^†^	2.68 (2.32 – 3.45)	<0.001
BOLD SI change (%)	1.25 (-2.38 – 7.89) * ^‡^	8.91 (4.95 – 12.78)^†^	14.08 (9.23 – 22.20)	<0.001

Data are presented as means ± standard deviation (95% confidence intervals) or median (interquartile range) as appropriate

Rest MBF corrected: rest MBF corrected for rate-pressure product (RPP), an index of myocardial oxygen consumption: MBF = (MBF/RPP) × 10^4^.

Stenosed: myocardial segments subtended by a >50% stenosed coronary artery.

Remote: myocardial segments subtended by arteries with minimal or no CAD.

Normal: myocardial segments in normal volunteers.

* p < 0.05 for comparison between stenosed and remote segments.

^†^ p < 0.05 for comparison between remote to ischemia and normal segments.

^‡^ p < 0.05 for comparison between stenosed and normal segments.

## Conclusion

T2-prepared SSFP 3 T BOLD imaging is feasible in the clinical setting and has good agreement with PET perfusion measurements for the detection of myocardial ischemia.
